# *In silico* bacteria evolve robust cooperaion via complex quorum-sensing strategies

**DOI:** 10.1038/s41598-020-65076-z

**Published:** 2020-05-25

**Authors:** Yifei Wang, Jennifer B. Rattray, Stephen A. Thomas, James Gurney, Sam P. Brown

**Affiliations:** 1 School of Biological Sciences, Georgia Institute of Technology, Atlanta, 30332 GA USA; 2 The Institute for Data Engineering and Science (IDEaS), Georgia Institute of Technology, Atlanta, 30332 GA USA; 3 Graduate Program in Quantitative Biosciences (QBioS), Georgia Institute of Technology, Atlanta, 30332 GA USA; 4 Center for Microbial Dynamics and Infection, Georgia Institute of Technology, Atlanta, 30332 GA USA

**Keywords:** Computational models, Bacterial evolution

## Abstract

Many species of bacteria collectively sense and respond to their social and physical environment via ‘quorum sensing’ (QS), a communication system controlling extracellular cooperative traits. Despite detailed understanding of the mechanisms of signal production and response, there remains considerable debate over the functional role(s) of QS: in short, what is it for? Experimental studies have found support for diverse functional roles: density sensing, mass-transfer sensing, genotype sensing, etc. While consistent with theory, these results cannot separate whether these functions were drivers of QS adaption, or simply artifacts or ‘spandrels’ of systems shaped by distinct ecological pressures. The challenge of separating spandrels from drivers of adaptation is particularly hard to address using extant bacterial species with poorly understood current ecologies (let alone their ecological histories). To understand the relationship between defined ecological challenges and trajectories of QS evolution, we used an agent-based simulation modeling approach. Given genetic mixing, our simulations produce behaviors that recapitulate features of diverse microbial QS systems, including coercive (high signal/low response) and generalized reciprocity (signal auto-regulation) strategists — that separately and in combination contribute to QS-dependent resilience of QS-controlled cooperation in the face of diverse cheats. We contrast our *in silico* results given defined ecological challenges with bacterial QS architectures that have evolved under largely unknown ecological contexts, highlighting the critical role of genetic constraints in shaping the shorter term (experimental evolution) dynamics of QS. More broadly, we see experimental evolution of digital organisms as a complementary tool in the search to understand the emergence of complex QS architectures and functions.

## Introduction

Many species of bacteria are highly social, investing in the secretion of multiple costly molecules in order to gain collective benefits. The benefits of microbial collective action are diverse, including extracellular digestion of complex molecules (via secretion of exo-enzymes^1^), access to limiting iron (via secretion of siderophores^2–4^), construction of defensive biofilms (via secretion of exopolysaccharide building blocks^5,6^ or anti-competitor toxins (via secretion of antibiotics and bacteriocins^7–10^).

Despite the shopping list of potential collective benefits, microbes like other social organisms face the challenge of identifying under what conditions costly investment in collective activity is going to return a net benefit. A striking commonality across multiple species of bacteria is the use of complex cell-cell signaling mechanisms known as quorum-sensing (QS) to control and coordinate the expression of social behaviors mediated by secreted factors^11^. Quorum-sensing bacteria secrete diffusible signal molecules, and respond to the accumulation of signal in their environment by changing global gene expression — increasing the relative rate of production of costly exo-enzymes, toxins and other secreted factors^12–16^.

The QS control of secreted factors has been long argued to allow bacteria to limit their costly investments in collective action to environments where a focal strain of bacteria are at high densities (‘quorate’) and therefore able to effectively modify their environment^17,18^, and it has more recently been demonstrated that cooperative exo-enzyme production is indeed more beneficial at higher densities^19^. Other theories suggest that QS allows bacteria to sense their physical environment such as the degree of containment or mass transfer^20,21^, the biochemical environment^22,23^ or through the use of multiple signals to simultaneously resolve both physical environment and population density^24^.

Bacteria also face potential uncertainty over the genotypic mix of their environment — are individuals exploiting a local environment as a single clone, or in the context of other strains or species? It is established that QS-controlled cooperative behaviors such as exo-enzyme production are vulnerable to social exploitation by ‘cheat’ strains that can reap the rewards without paying the costs of secretions, and that this vulnerability can be mitigated by positive genetic assortment/kin selection^25–28^, and also by the coupling of directly beneficial traits under QS control^23,29^. Other studies have explored how the architecture of QS allows individuals to sense and strategically respond to variations in the genotypic environment^30–33^, and on a longer evolutionary time-scale how the tuning and architecture of QS is itself potentially shaped by enduring patterns of genetic conflict^16,25,30,34,35^.

The complexity of QS also raises the possibility of additional dimensions of social conflict beyond the extent of costly exo-product production. In common with any communication system, QS is the product of the coupled evolution of a signal and a response strategy^36^; implying that the return on investment in signaling depends on the response strategy (signal affinity), and vice versa. This coupling highlights that to hit a defined density (or diffusion, etc) threshold there are multiple signal/response equilibria: for example a high signaling/low response ‘shouting and deaf’ equilibrium can be equally effective for a clonal population detecting a threshold as low signaling/high response ‘whispering and attentive’ equilibrium^25^. These alternate equilibria are not equivalent however in a non-clonal context; a high signal/low response strategist will act to coercively induce greater cooperative returns whenever mixed with a low signal/high response strategist, leading to their predicted dominance under conditions of intermediate genetic mixing^25^.

The complexity and potential multi-functionality of QS has lead to a growing number of experimental studies designed to experimentally determine the functional roles of QS across multiple bacterial systems^17,19,20,24,30,32,37–43^. Across these experimental systems, there is evidence that QS systems can limit cooperative investments to high density^19^, low mass transfer^21,44^ and clonal^30,32^ environments — in some cases these responses have demonstrated fitness advantages^19,30,32^. While consistent with theoretical predictions, these results cannot separate whether these functional roles were the drivers of QS evolution, or simply fortuitous byproducts or ‘spandrels’^45^ of a complex system driven by other ecological pressures.

The challenge of separating drivers of adaptation from ‘spandrel’ properties is particularly hard to address using extant bacterial species with complex QS architectures that have evolved under ecological conditions that we scarcely understand. In this context, studying evolution using digital organisms with defined ecologies can provide new insights into the general properties of evolution^46^. Previous work has established two major simulation platforms to conduct *in silico* evolutionary experiments, Avida and Aevol. Each digital organism in Avida is a self-contained computing automaton that is capable of self-replicating, mutating, and competing for limited resources — the central processing unit (CPU) cycles^47^. Avida has been employed to address diverse general evolutionary principles^48–50^, but with less focus on social evolution (but see e.g.^51^). In contrast to Avida, Aevol has explicitly modeled the genetic architecture for individuals^52^. Aevol has also been widely used in *in silico* studies of cooperation^53–55^.

While Aevol introduced an explicit genetic architecture, this platform lacks quorum-sensing control of gene expression. To address this limitation we previously developed an agent-based evolutionary model incorporating quorum-sensing^24^. Our previous analysis demonstrated that QS architectures can evolve to resolve defined environmental challenges, given clonal evolution. In the current study, we relax the assumption of clonality, and extend the dimensions of evolution. Specifically, we study the joint evolution of multiple QS component traits (basal signal production, cooperative response, signal auto-regulation) under a range of conditions of environmental heterogeneity (variable densities and variable genetic mixing among groups). Finally, we contrast our results with bacterial QS architectures that have evolved under unknown ecological contexts.

## Results

### The quorum sensing coordination game in a clonal context

**Figure 1:**
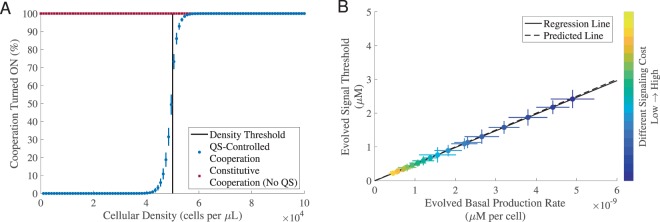
Signal production rate and threshold response co-evolve in a quorum sensing co-ordination game. We evolved 5,000 initially identical genotypes for 5,000 generations in a patchy, variable density environment (see *Methods Summary* and *Supplemental Material* for more details). In all simulations, the population was clonal, i.e., *G* = 1, the cost of cooperation was fixed and there was no auto-regulation. (**A**) We used a fixed cost of signaling, and recorded the percentage of evolved individuals who turned on cooperation in 100 testing environments where the cellular density varied from 10^1.5^ to 10^5^ cells per *μL*. Note constitutive individuals, by definition, will always turn on cooperation in all environments as shown by the red dots. The vertical black line indicates the pre-set density threshold *N*_*Th*_, i.e., the median cellular density across 100 testing environments (5.0016 × 10^4^ cells per *μL*). (**B**) Varying the cost of signaling (*C*_*sig*_ ∈ [5 × 10^8^, 100 × 10^8^]; step size: 5 × 10^8^) predictably impacts the balance of signal production and response. Each dot represents the evolved mean results (averaged over the last 50 generations). The color-bars indicate different values of *C*_*sig*_ from low signaling cost (dark blue) to high singling cost (bright yellow). The solid black line is the regression line fitted using the generalized linear model with a normal distribution: *R*^2^ = 1, *F*-test, *p* = 5.511 × 10^−50^. The dashed black line is the predicted line calculated by *S*_*Th*_ = *pN*_*Th*_/*u*, where *u* = 10^−4^ *μL*/*s*. The horizontal and vertical error bars represent the standard deviation over 30 replications. The remaining parameters used in the simulations can be found in *Supplemental Material*, Table S1. Note that given the computational complexity, in most cases, we only evolved the population for 5,000 generations. Evolving for a longer time will not change the results qualitatively. An example of evolution trajectories from (**B**) can be found in *Supplemental Material*, Fig. S1.

We begin by defining a cooperative trait with a threshold density dependent benefit (see *Methods Summary* and *Supplemental Material*), and illustrate that the joint evolution of signal production (*p*) and signal threshold to response (*S*_*Th*_) can tune individual cooperative behavior to solve the ‘density sensing’ problem (Fig. 1A).

Our analyses show that bacteria solve this problem by resolving a co-ordination game between signal and response strategies. Given our model of extracellular signal dynamics in the absence of auto-regulation (Eq. 1, see *Methods Summary*), we can see that for a given stationary density *N*, signal concentration *S* will equilibrate to S^*^ = *pN*/*u*, where *u* is the rate of environmental signal degradation. Given a critical density threshold (for cooperation to pay) of *N*_*Th*_, we can define the critical signal threshold *S*_*Th*_ = *pN*_*Th*_/*u*, so that signal will trigger cooperation if S^*^ > *S*_*Th*_. Together, this implies that QS bacteria will cooperate when *pN*/*u* > *S*_*Th*_. Given that both *p* and *S*_*Th*_ are evolutionary variables, the joint evolution of both traits forms a coordination game — the optimal value of signal production *p* depends on *S*_*Th*_, and vice versa. In Fig. 1B, we illustrate the nature of this coordination game by varying the cost of signaling. As shown in Fig. 1B, as the cost of signaling increases, the basal signaling rate *p* evolves to a lower intensity. As a consequence, the signal threshold *S*_*Th*_ decreases accordingly in order to maintain resolution of density threshold *N*_*Th*_, as predicted by *S*_*Th*_ = *pN*_*Th*_/*u* (dashed line in Fig. 1B). Previous mathematical (game-theoretic) analyzes of QS evolution in a clonal context indicates that signal costs will drive signal/response evolution towards the lowest production rate that is consistent with reliable communication — described previously as a ‘conspiratorial whisper’^25^. Our agent-based simulations all display the predicted transients, with the fastest approach driven by the largest signal costs. In *Supplemental Material*, Figs S2A, B (also see *Supplemental Material*, Fig. S3) we illustrate that the evolved coordination of signaling *p* and response *S*_*Th*_ is also dependent on environmental factors governing signal decay rate and noise. Together, these results illustrate that clonal bacteria can coordinate their behaviors to adjust their signal production rate and their responsiveness to signal, and this QS-controlled cooperation is superior to constitutive cooperation, given density fluctuations and positive density dependence.

### Genetic mixing can lead to coercive strategies

**Figure 2:**
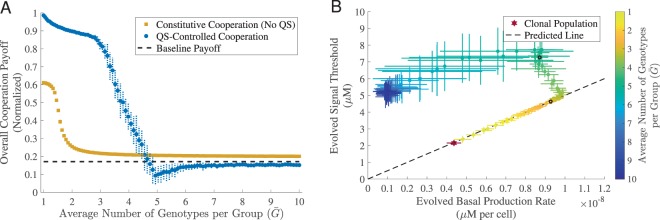
Coercive QS strategies maintain cooperation in genetically mixed populations. For both constitutive cooperation and QS-controlled cooperation, we used a fixed cost of cooperation. We also used a fixed cost of signaling in QS-controlled cooperation. In all cases, we evolved a population of 5,000 individuals for 5,000 generations. Each dot represents the evolved mean results (averaged over the last 50 generations) for different average number of genotypes per group $\bar{G}$. The horizontal and vertical error bars represent the standard deviation over 30 replications. (**A**) Overall cooperation payoff (evolutionary robustness). The dashed black line represents the baseline payoff. (**B**) Co-evolved production rate and signal threshold for QS-controlled cooperation. The dashed black line is the predicted line calculated by *S*_*Th*_ = *pN*_*Th*_/*u*, where *u* = 10^−4^ *μL*/*s* and *N*_*Th*_ is the median cellular density across 100 testing environments (5.0016 × 10^4^ cells per *μL*). The red star and black dots represent represent three specific populations with varying relatedness (*G* = 1, $\bar{G}\approx 2.13$ and $\bar{G}\approx 4.07$) that are used to examine spandrel function in Fig. 5. Examples of evolution trajectories as well as population diversity from (**B**) can be found in *Supplemental Material*, Fig. S5, S6, respectively. The remaining parameters used in the simulations can be found in *Supplemental Material*, Table S1.

Next, we ask what are the effects of genetic mixing on the evolution of QS-controlled cooperation? To explore this question, we performed simulations where we varied the average number of genotype founders per local population $\bar{G}$, and contrasted QS-controlled and constitutive cooperation (Fig. 2A). In the clonal limit ($\bar{G}\to 1$), we see the efficiency benefit of matching cooperative behavior to environment compared to constitutive cooperation, as illustrated in Fig. 1A. As the degree of genotypic mixing increases, the levels of constitutive cooperation are fast diminishing. In contrast, cooperation is more robust to increased genetic mixing when controlled by QS. Note that the average payoff of QS-controlled cooperation is below the baseline, for high levels of $\bar{G}$. This is because of persistent costs of low levels of signaling, maintained due to selection-drift balance.

To understand the greater robustness of QS controlled cooperation we examined the joint evolution of the component signal and response traits under different conditions of genetic mixing (Fig. 2B, also see *Supplemental Material*, Fig. S4A). Consistent with earlier theory^25^, we found that clonality selected for ‘whispering and attentive’ strategies, coupling low signaling with low response thresholds. Conversely, under moderate genetic mixing, theory predicts ‘coercive’ strategies with higher investments in signal production and lower responsiveness^25^. Consistent with this theory, we found the evolution of ‘coercive’ strategies — capable of inducing greater cooperative responses from their less coercive ancestors when sharing a local population. For low to intermediate levels of genetic mixing ($\bar{G}=1$ to 3), we see that the evolved genotypes stay close to the functional constraint (*pN*/*u* = *S*_*Th*_, dashed line in Fig. 2B), which implies that they are able to effectively identify the density threshold when working as a solitary clone (as in Fig. 1A). However, in the event of a mixed sub-population the genotype with the higher signal and response threshold will act as a conditional cheat by inducing greater cooperative investment from its partner. As genetic mixing is further increased, the probability of ever experiencing a clonal environment is diminished (e.g. for $\bar{G}=3$, the per sub-population probability of clonality is ~0.17), and therefore selection on clonal efficacy is relaxed. In these low relatedness contexts, we see evolutionary trajectories towards simple cheating strategies, captured by high response thresholds and low/drifting signal production (Fig. 2B).

To explore how genetic constraints affect QS-controlled cooperation, we also investigated the overall cooperation payoff when one trait was genetically constrained to be constant. When only evolving the response threshold (holding signal production constant and non-zero), the overall cooperation payoff is rapidly lost when $\bar{G} > 2$ (*Supplemental Material*, Fig. S7). These ‘response-evolving’ populations can perform effective QS regulation in a clonal context (*G* = 1), but they are more prone to cheat takeover given genetic mixing compared to the joint-evolving populations (Fig. 2A), as they cannot evolve coercive strategies. In contrast, the ‘signal-evolving’ populations (with genetically fixed response thresholds) illustrate an example of a genetic constraint driving increased cooperative robustness (*Supplemental Material*, Fig. S7).

### Auto-regulation sustains QS-controlled cooperation under high genetic mixing

**Figure 3:**
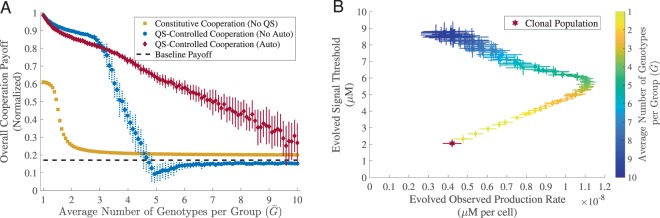
Auto-regulation extends the window of QS-controlled cooperation to greater genetic mixing. For both constitutive cooperation and QS-controlled cooperation, we used a fixed cost of cooperation. We also used a fixed cost of signaling in QS-controlled regimes. In all cases, we evolved a population of 5,000 individuals for 5,000 generations. Each dot represents the evolved mean results (averaged over the last 50 generations) for different average number of genotypes per group $\bar{G}$. The horizontal and vertical error bars represent the standard deviation over 30 replications. (**A**) Overall cooperation payoff. The black dashed line represents the baseline payoff. (**B**) Co-evolved production rate and signal threshold for QS-controlled cooperation with auto-regulation. The red star represents the clonal population (*G* = 1). Examples of evolution trajectories as well as population diversity from (**B**) can be found in *Supplemental Material*, Fig. S8, S6, respectively. The remaining parameters used in the simulations can be found in *Supplemental Material*, Table S1.

Recent theory and experimental work has suggested that auto-regulation (specifically, a positive feedback loop between signal response and signal production^22,56^) helps to maintain QS-controlled cooperation by increasing phenotypic assortment^32^. To investigate the role of auto-regulation in our simulation framework, we repeated the analyses of the previous section with the addition of a third evolutionary dimension, auto-regulation ratio *r* (the ratio of maximally induced production to baseline signal production (Eq. 2, see *Methods Summary*)). We first compared the overall cooperation payoff of QS-controlled cooperation with auto-regulation against QS-controlled cooperation without auto-regulation and constitutive cooperation. From Fig. 3A, we see that auto-regulation QS enhances the evolutionary robustness of cooperation in the face of medium to high levels of genetic mixing.

To begin to decipher the mechanisms of greater resilience of QS with auto-regulation, we again examined the joint evolution of the three component traits, *p*, *S*_*Th*_ and *r* (Fig. 3B, and also see *Supplemental Material*, Fig. S4B). In *Supplemental Material*, Fig. S9, we see that the evolved auto-regulation ratio *r* is close to 1 in the clonal context (i.e. doubling of total signal production under maximal auto-regulation, compared to baseline), suggesting there is some benefit to auto-regulation in a clonal context. In contrast, under conditions of genetic mixing auto-regulation evolves to higher levels, peaking at 8 (*Supplemental Material*, Fig. S9) for intermediate levels of mixing. In Fig. 3B (also *Supplemental Material*, Fig. S9) we plot total signal production (i.e. a composite of baseline and auto-regulation behavior) against response threshold, and similarly to Fig. 2B we see the signature of a coercive escalation of signal and response threshold as genetic mixing increases. However, in contrast to Fig. 2B we now see that even at our highest levels of genetic mixing, there is still sufficient signaling and responsiveness (Fig. 3B and *Supplemental Material*, Fig. S9) to maintain cooperative rewards above baseline (Fig. 3A).

To further challenge quorum sensing controlled cooperation, we introduced constitutive (and immutable) cheats at a certain rate in every generation (constitutive cheats have zero signal production and maximal signal threshold. See *Supplemental Material* for more details). As can be seen from *Supplemental Material*, Fig. S10 (also see *Supplemental Material*, Fig. S11), the evolution of auto-regulation also protects against challenge with constitutive cheats.

### Generalized reciprocity protects QS-controlled cooperation from exploitation by cheats

**Figure 4:**
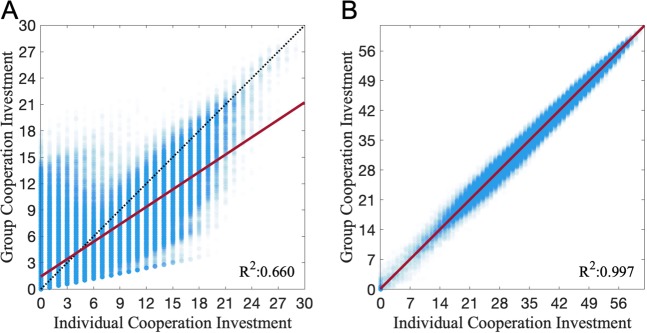
Generalized reciprocity facilitates QS-controlled cooperation. For fixed costs of cooperation and signaling with the number of mixing genotypes *G* = 5, we collected 5,000 same initial genotypes and evolved them for 5,000 generations with no auto-regulation (**A**) and auto-regulation (**B**). We recorded the individual and group mean investment for cooperation at the last generation over 100 replications. Each blue dot represents an individual’s investment against its group mean investment. The red lines are the regression lines fitted using the generalized linear model with a normal distribution. The analysis of covariance shows there is a significant difference between the slope of no auto-regulation in (**A**) and the slope of auto-regulation in (**B**) (*F*-test, *p* = 0.000). Similar results varying *G* can be found in *Supplemental Material*, Fig. S12. The remaining parameters used in the simulations can be found in *Supplemental Material*, Table S1.

QS-controlled cooperation is vulnerable to social exploitation by cheats who are not paying individual costs but can benefit from others producing public goods^25–27^. However, in the last section we have shown that auto-regulation plays an important role in protecting cooperation. To build a mechanistic understanding of why QS-controlled cooperation with auto-regulation is more robust, we measured the phenotypic assortment between individual and group cooperative investment for different levels of genetic mixing. Fig. 4A shows that in the case of *G* = 5 (five genotypes per sub-population) and the absence of auto-regulation, the relationship between the cooperative behavior of an individual (x-axis) and of its group (y-axis) is positive but with substantial variation. In contrast, the introduction of auto-regulation (Fig. 4B) produces a much tighter relationship between individual and group levels of cooperation. Together, Fig. 4 (also see *Supplemental Material*, Fig. S12) illustrates that positive auto-regulating bacteria are better able to coordinate their cooperative investment at a group level, and therefore reduce the degree of exploitative mismatches between focal individuals and other members of the group (see^32^).

Consistent with Fig. 4, a levels of selection partitioning using a two-level Price equation (see *Supplemental Material* for more details) illustrates that auto-regulation acts to dramatically reduce within-group selection for cheats (see *Supplemental Material*, Figs S13, 14).

### Adaptation to density sensing can produce diffusion-sensing spandrels

In the beginning of this paper, we outlined a general and experimentally challenging question — how can we infer adaptive function using extant organisms with unknown environmental histories? In the case of QS bacteria, we can experimentally identify function (for instance, the ability to respond positively to density^19^), but even the observation of an *in vitro* fitness benefit^19^ does not preclude the possibility of a fortunate ‘spandrel’^57^. In the current study we have evolved QS in digital organisms under a defined (density sensing) ecological challenge, so we can now assess their functionality in the context of environmental challenges outside of their evolutionary history — do we see spandrels?

**Figure 5:**
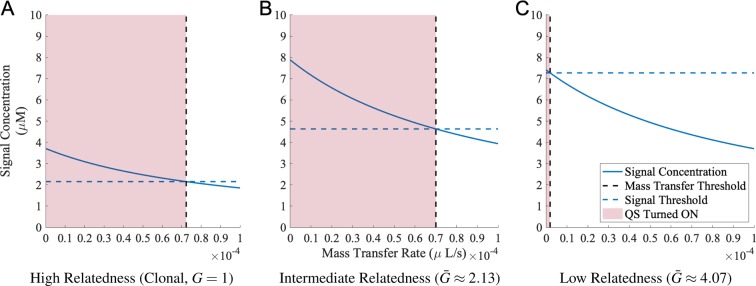
QS-controlled cooperation can evolve ‘diffusion sensing’ as spandrels. From previous simulations as shown in Fig. 2B, we took three evolved strains: (**A**) Clonal strain (*G* = 1, *p* = 4.37 × 10^−9^ *μM*/*s* and *S*_*Th*_ = 2.15 *μM*); (**B**) Strain with intermediate relatedness (*λ*_*G*_ = 2, $\bar{G}\approx 2.13$, *p* = 9.27 × 10^−9^* μM*/*s* and *S*_*Th*_ = 4.63* μM*); (**C**) Strain with low relatedness *(λ*_*G*_ = 4, $\bar{G}\approx 4.07$, *p* = 8.71 × 10^*−*9^ *μM*/*s* and *S*_*Th*_ = 7.27* μM*). We then fixed the cellular density (*N* = 8.5 × 10^4^ cells per *μL*), and introduced the mass transfer rate (*m* ∈ [0, 10^*−*4^ *μL*/*s*]). The solid blue lines represent the signal concentrations calculated by *p* × *N*/(*u* + *m*) (*u* = 10^*−*4^ *μL*/*s*). The dashed blue lines are signal thresholds of the evolved strains, and dashed black lines are mass transfer rate thresholds (when S^*^ = *S*_*Th*_) for the strain below which the cooperation is turned on (red areas).

To assess non-evolved functionality we took three previously evolved strains (the red star along with two black dots in Fig. 2B) and assessed their performance in a novel environment. Specifically, we fixed bacterial density to a high (quorate) level (*N* = 8.5 × 10^4^ cells per *μL*) and introduced a new dimension of environmental variation — mass transfer (e.g. diffusion or advection). We capture mass transfer as an additional signal loss term at rate *m*. As a result the equilibrium extracellular signal concentration is now governed by a new equation *p* × *N*/(*u* + *m*). Fig. 5 illustrates the performance of the three evolved strains at high density and under varying mass-transfer regimes, illustrating that the two strains can qualitatively perform ‘diffusion sensing’ (turning QS genes ‘ON’ only when mass-transfer is limited). The clonal and intermediate relatedness strains use different production and signal threshold parameters to achieve a near-identical mass-transfer threshold (Fig. 5A,B). This reflects the fact that both strains evolved to optimize for the same density threshold and also evolved along the functional constraint linking production and signal response (see dashed line, Fig. 2B). In contrast, the low relatedness strain has evolved a disproportionately high response threshold (Fig. 2B) and in consequence is more sensitive to signal losses due to mass-transfer (Fig. 5C). While these strains produce differing quantitative responses to variation in diffusion or advection, we know through our simulation design that none of these strains have been evolutionarily tuned to solve any specific mass-transfer challenge. Therefore these strains are liable to make quantitative mistakes under a novel diffusion-sensing challenge.

## Discussion

In this study we examined the evolutionary dynamics of quorum sensing traits in an *in silico* system, to remove the complexities of experimental model systems that have evolved under diverse and largely unknown ecological contexts. Stripping away the system-specific complexities of quorum-sensing highlights that QS is at base a co-ordination game, where the reward for a particular signaling strategy depends on the prevailing strategy of response and vice versa^58,59^. Under the defined context of our *in silico* environments, populations that exploit environments clonally can jointly tune signal and response traits to effectively resolve and respond to variation in local sub-population density, triggering cooperative investments only in sub-populations where density is above a critical threshold (Fig. 1). The introduction of genetic mixing ($\bar{G} > 1$ founder per sub-population) led to a broader array of strategies including ‘coercion’ (higher signal, higher response threshold, Fig. 2) and ‘generalized reciprocity’^60–62^ (positive auto-regulation, Fig. 3) that both independently and in conjunction contribute to the maintenance of QS regulated cooperative investments, in the face of cheats.

The description of higher signal/higher response threshold strains as ‘coercive’ is motivated by their ability to force greater degrees of cooperative response when mixed with ancestral lower response strains, while also maintaining their ability to work effectively as a clone. Note that the coercion differs from simple cheating due to the presence of an active manipulative behavior (here, the production of additional signal to induce greater cooperative responses from interactants). Kentzoglanakis *et al*. present a conceptually related *in silico* evolution model to describe a distinct biological phenomenon^63^: the evolution of plasmid intracellular copy-number control by the joint action of plasmid encoded trans-acting replication inhibitors (the signal trait), and the binding affinity of the inhibitor targets (the response trait). The authors demonstrated that the joint evolution of the signal and response trait generates increased collective efficiency (in this case, optimization of plasmid number within cells), and interpreted this model in the context of the evolutionary ‘policing’ literature^64,65^, with the repressor/signal interpreted as a ‘policing’ trait, and the target affinity/response trait interpreted as a critical and joint-evolving ‘obedience’ trait. In both the plasmid and QS contexts, we see the potential for similar co-evolutionary runaways towards increasing coercion (high signal, low response equilibria) under conditions of increased genetic mixing (Fig. 2 and also see Fig. 4 in^66^). However, as genetic mixing increases this coercive peak in signaling (policing) fails due to a collapse in obedience/response (Fig. 2). The resulting hump in signal investment with increased genetic mixing is predicted by a simple analytical game theory model^25^ and now has support from two distinct simulation models built with very different biological motivations (this study and^63,66^), which raises the challenge of why this result has been difficult to pin down experimentally, despite explicit attention^62^. Later in the discussion we return to this point in a general overview of the empirical context, but in short, it appears that the genetics of auto-regulation present an effective mechanistic block to the elaboration of coercive strategies.

One of the key hallmarks of many (but not all) QS regulatory architectures is signal auto-regulation, where signal response is coupled to increase the signal production^40,67–70^, leading to increased synchrony across individual cellular responses^56^. To explore the evolutionary role of auto-regulation in our system, we added auto-regulation as a third evolving trait, and found that this additional evolutionary dimension led to a further increase in the robustness of QS controlled cooperation (Fig. 3). In the evolved auto-regulation lineages we found a stronger degree of phenotype matching (assortment) between individuals and their group (Fig. 4), demonstrating that positive feedback control of signal production allows individuals to tune their cooperative behavior to their social environment. This result is consistent with a recent experimental paper on *P. aeruginosa*, which demonstrated that *P. aeruginosa* can facultatively tune its per-capita cooperative investment to the proportion of wildtype cooperators in its local group, in a manner that will promote the maintenance of cooperation^32^. Allen *et al*. described this behavior as an example of generalized reciprocity, highlighting that by encoding a simple rule of ‘cooperate when with cooperators’ bacteria can increase the robustness of cooperation and the regulatory architectures that control cooperation^32^.

In the simple environmental and genetic world of our *in silico* bacteria, populations readily evolve complex strategies of coercion and generalized reciprocity. While generalized reciprocity has been reported for *P. aeruginosa*, coercion has been far more elusive, despite direct experimental evolution tests^62^. Popat *et al*. experimentally evolved *P. aeruginosa* under conditions of high and low genetic mixing, and found that under conditions of intermediate and low genetic mixing, the level of signal production only went down (alongside response); there was no peak in coercion^62^. One possible account for this disconnect with our simulations is that on the ~1 month timescale of experimental evolution the evolutionary dynamics are constrained by the genetic mechanisms of auto-regulation: The easiest solution to reduce signal response is to mutate the signal receptor (in *P. aeruginosa*, this is frequently achieved by Δ*lasR* mutations) which has the pleiotropic consequence of also largely abolishing signal production.

This argument suggests that coercive strategies are more likely to be evolvable on short timescales in bacteria without strong auto-regulatory constraints, such as *V. cholerae*^71^ (but see^72^). In our main text results all traits could independently evolve, and thus both generalized reciprocity (signal auto-regulation) and coercion (high signal/low response) are accessible simultaneously. In *Supplemental Material*, Fig. S7, we introduced simple genetic constraints (constraining the evolution of one trait and allowing others to freely evolve) and found substantial shifts in evolutionary trajectories, either helping (with a fixed response, see blue dots in *Supplemental Material*, Fig. S7) or harming (with a fixed signal, see yellow dots in *Supplemental Material*, Fig. S7) the maintenance of cooperation depending on genetic details.

The existence of a genetic constraint does not imply that over longer time-scales the constraint is immutable. Take for example the constraint imposed by *lasR* co-regulation on the trajectories of signal production and signal threshold in *P. aeruginosa*. Sandoz *et al*. reported two *lasR* mutants (*lasR*5 and *lasR*8) that displayed near-wildtype levels of signal production but with lower level of signal response^27^. In principle, it is possible that signal production and response could evolve independently in *P. aeruginosa* by separately targeting steps that are downstream of *lasR*, for instance targeting multiple promoter sites to separately tune the impact of LasR on signal synthase and cooperative effector genes. Gurney *et al*.^33^ recently demonstrated using experimental evolution that *P. aeruginosa* can rewire its response to multiple signal inputs in order to escape ancestral genetic constraints on social behaviors — in this example, through the evolution of novel cheating strategies to escape pleiotropic constraints termed ‘metabolic incentives to cooperate’^29^.

It is important to stress that the emergence of recognizable QS features (auto-regulation, coercive signaling strategies) in our evolutionary simulation does not imply that the selective conditions in our simulation necessarily match the (unknown) selective conditions shaping bacterial evolution in ‘the wild’. In our ‘*in silico*’ evolution, we know by definition the ecological challenges that bacteria are facing. Specifically, we defined a density threshold for the rewards of turning on cooperation and showed that bacteria can evolve strategies that are adaptations to ‘density sensing’. However, as a result we inevitably also evolve spandrels (a byproduct of adaptive selection, see^45^). For example, we illustrate that our evolved bacteria can perform a ‘diffusion sensing’ role (Fig. 5) to differentiate mass transfer regimes^20^, despite never experiencing this challenge. On the other hand, it is possible that if we set the environmental challenges to ‘diffusion sensing’, we will evolve ‘density sensing’ as spandrels (or exaptation). The ability to precisely define and control the environment of adaptation and the genetic constraints of the ancestor suggest that *in silico* bacteria are a fruitful model for the study of adaptation and exaptation in quorum-sensing bacteria, opening a window into the mapping between defined selective environments and QS phenotypes.

## Methods Summary

**Figure 6:**
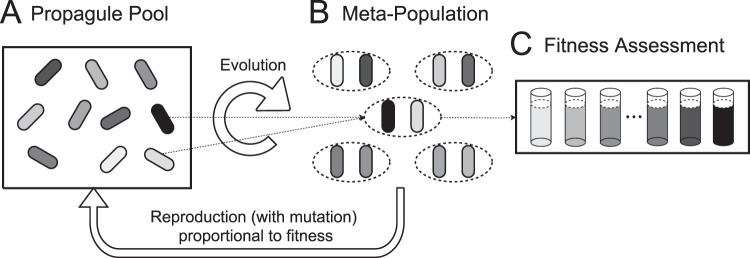
*In silico* evolution of quorum sensing. (**A**) The propagate pool at time zero is a strategically uniform population of *in silico* individuals. (**B**) In each generation, individuals are distributed into locally interacting sub-populations depending on the condition of genetic mixing (defined by number of founding cells per sub-population — two founders per sub-population in this illustration). (**C**) The fitness of all individual founders is evaluated across a range of testing environments (tested in 100 different population densities picked uniformly from 10^1.5^ to 10^5^ cells per *μL*). Then, individuals are selected proportionally to their payoffs for clonal reproduction but are subject to mutation in their quorum sensing traits (signal production, threshold to response, and in some simulations, auto-regulation). Finally, the offspring pool with the same size as the initial propagate pool was formed for the next generation.

In our agent-based *in silico* evolution experiments, we first consider two evolving traits, basal production rate (*p*) and signal response threshold (*S*_*Th*_). In subsequent simulations, we introduce an additional evolving trait, the auto-regulation ratio (*r*), the ratio of fully induced to basal signal level. These co-evolving traits combine to govern individual cell decisions to turn on or off cooperation in different environmental conditions. Populations are subject to selection based on individual cooperation payoffs (incorporating costs of cooperation and signaling, and environment-dependent benefits), and evolve for a fixed number of generations as shown in Fig. 6 (see *Supplemental Material* for more model implementation details).

The fitness of each genotype in each sub-population is assessed across a spectrum of potential bacterial carrying capacities (see Fig. 6). For carrying capacity (at density *N*), signaling and resulting cooperative responses are described by an ODE governing extracellular signal concentration. Specifically, we consider the following two scenarios of signal dynamics for QS-controlled cooperation in absence (Eq. 1) and presence (Eq. 2) of auto-regulation, respectively.

1 $$\frac{dS}{dt}=pN-uS,$$

2 $$\frac{dS}{dt}=p\left(1+r\frac{S}{K+S}\right)N-uS,$$

where *S* is the local signal concentration, *t* is time, *N* is the stationary phase cell density, *p* is the basal signal production rate, *r* is the ratio of auto-regulation production to basal signal production, *K* is the half concentration value, and *u* is the signal decay rate. We assume that signal concentration rapidly equilibrates to S^*^ (see *Supplemental Material*), and use this value to determine individual cooperation. Individuals will turn on their cooperative phenotype only when the local signal concentration is higher than the individual response threshold (S^*^ > *S*_*Th*_). Both signaling and cooperation are costly to individuals, but they benefit from cooperation only when the local cellular density is above a certain threshold *N*_*Th*_. Therefore cost-effective cooperative behavior is dependent on the effective tuning of QS to identify an underlying density threshold. For constitutive cooperation (No QS), individuals will always turn on cooperation regardless of local signaling environment — they do not have the ability to make social informed choices.

### Accession codes

The Julia source code of QS simulations can be downloaded here: https://bit.ly/2u3OcSM.

## Supplementary information

Supplemental Material.

Evolution trajectory of population with high relatedness (No Auto).

Evolution trajectory of population with high relatedness (Auto).

Evolution trajectory of population with intermediate relatedness (No Auto).

Evolution trajectory of population with intermediate relatedness (Auto).

Evolution trajectory of population with low relatedness (No Auto).

Evolution trajectory of population with low relatedness (Auto).
