# Author Correction: *In silico* bacteria evolve robust cooperation via complex quorum-sensing strategies

**DOI:** 10.1038/s41598-020-68366-8

**Published:** 2020-07-15

**Authors:** Yifei Wang, Jennifer B. Rattray, Stephen A. Thomas, James Gurney, Sam P. Brown

**Affiliations:** 1 School of Biological Sciences, Georgia Institute of Technology, Atlanta, GA 30332 USA; 2 The Institute for Data Engineering and Science (IDEaS), Georgia Institute of Technology, Atlanta, GA 30332 USA; 3 Graduate Program in Quantitative Biosciences (QBioS), Georgia Institute of Technology, Atlanta, GA 30332 USA; 4 Center for Microbial Dynamics and Infection, Georgia Institute of Technology, Atlanta, GA 30332 USA

Correction to: *Scientific Reports* 10.1038/s41598-020-65076-z, published online 25 May 2020

The original version of this Article contained an error in the title of the paper, where the word “cooperation” was incorrectly given as “cooperaion”. This has now been corrected in the PDF and HTML versions of the Article and in the accompanying Supplementary Information file.

