# Supplementary material for: In silico bacteria evolve robust cooperaion via complex quorum-sensing strategies
Source: Sci Rep. 2020 May 25;10:8628. doi: 10.1038/s41598-020-65076-z (PMC7248119; doi:10.1038/s41598-020-65076-z)
Supplement: Supplementary file 1 — Supplemental Material. [file 41598_2020_65076_MOESM1_ESM.pdf]

In silico bacteria evolve robust cooperaion via complex quorum-sensing strategies

Yifei Wang[1,2,4,*], Jennifer B. Rattray[1,4], Stephen A. Thomas[1,3,4], James Gurney[1,4], Sam P. Brown[1,4,*]

1. School of Biological Sciences, Georgia Institute of Technology, Atlanta, 30332 GA, USA

2. The Institute for Data Engineering and Science (IDEaS), Georgia Institute of Technology, Atlanta, 30332 GA, USA

3. Graduate Program in Quantitative Biosciences (QBioS), Georgia Institute of Technology, Atlanta, 30332 GA, USA

4. Center for Microbial Dynamics and Infection, Georgia Institute of Technology, Atlanta, 30332 GA, USA

* Corresponding Authors: yifei.wang@gatech.edu (Y.W.) and sam.brown@biology.gatech.edu (S.P.B.)

Supplemental Material

Simulation model

In the *in silico* evolution, we consider two evolving traits, basal production rate (p) and signal response threshold (S_{Th}) for simulations in the absence of auto-regulation, whereas we introduce an additional evolving trait, auto-regulation ratio (r) for simulations including the auto-regulation mechanism. Each individual in the population pool has a single genotype which consists of those two or three evolving traits. The individuals can make their own decisions to turn on or off cooperation as a function of signal mediated interactions, which in turn depend on the physical and social environment. Specifically, the evolution process is described in *Main Text*, Figure 6:

1) Total N_{pop} genotypes with same initial traits (same p_{init}, $S_{Th_{\text{init}}}$ and r_{init}) were generated to form a population pool.

2) A certain number of genotypes (G, drawn from zero-truncated Poisson distribution, unless otherwise specified) were randomly selected (with replacement) from the population pool and form a mixed sub-population.

3) For each of N_{env} sub-population testing environments, the signal concentration in the mixed sub-population can be calculated as S^* using Eq. (S2) (or Eq. (S7) for auto-regulation case).

4) Each genotype in the mixed sub-population was evaluated for its overall cooperation payoff separately across all sub-population testing environments (where the cellular density was varied) using Eq. (S3) (or Eq. (S8) in auto-regulation case): Each individual paid for its own cost for signaling and the cost of cooperation, if any, but only gained a benefit when the number of cooperators in sub-population were greater than a certain threshold, N_{Th}.

5) Repeat 2) to 4) until the same size of population pool was formed.

6) All individuals were selected (with replacement) from the population pool to reproduce with a probability proportional to their overall cooperation payoff.

7) All evolving traits (p, S_{Th} and r) of the offspring were subject to mutation at rates λ_p, $\lambda_{S_{Th}}$ and λ_r with standard deviations SD_p, $SD_{S_{Th}}$ or SD_r for different traits. Specifically, for each evolving trait, the actual number of individuals selected for mutation was drawn from a Poisson distribution with the mean being λ_p, $\lambda_{S_{Th}}$ and λ_r, respectively. The mutation operation was done by adding a value of

$N(0, SD)$ to the original trait value, where $N(0, SD)$ is the normal distribution with a mean 0 and a standard deviation SD to be SD_p, $SD_{S_{Th}}$ or SD_r for different traits.

8) Repeat 2) to 7) until $Gen_{\max}$ generations were reached.

Model assumptions

For the computational models presented in the paper, we assume that:

1) A single signal type exists.
2) In the propagule pool, the cellular heterogeneity can be obtained via mutation and selection.
3) Each sub-population is founded by a defined number of founding cells G (1 to 10), picked randomly from the propagule pool of the previous generation.
4) For each sub-population, the expected fitness of each founding cell is assessed across a range of testing environments (cellular density varied from $10^{1.5}$ to 10^5 cells per μL) where the founding cells immediately grow until the total sub-population reaches a stationary phase density defined by its current testing environment.
5) In each testing environment, the sub-population forms a closed system, i.e., no mass transfer.
6) For a given testing environment, an individual can be rewarded with a benefit for cooperation only if the number of cooperators is greater than the defined threshold (N_{Th}).
7) The signal concentration in each testing environment rapidly reaches equilibrium estimated by Eq. (S1) or Eq. (S6), depending on whether invoking the auto-regulation mechanism.

Computational model of quorum sensing without auto-regulation

The computational model of signal dynamics for quorum sensing without auto-regulation is given as below:

$$\frac{dS}{dt} = pN - uS, \tag{S1}$$

where S is the local signal concentration, t is time, N is the local cell density, p is the basal signal production rate, and u is the signal decay rate. The equilibrium of Eq. (S1) is given by:

$$S^* = \frac{pN}{u}. \tag{S2}$$

In the absence of the auto-regulation mechanism, the individual genotype's overall cooperation payoff across all sub-population testing environments is assessed as follows:

$$F_i = B_0 + B_{coop} \sum_j H_{B_{ij}} - C_{coop} \sum_j H_{C_{ij}} - C_{sig} p_i, \tag{S3}$$

where i ($i = 1, 2, \cdots, N_{pop}$) represents an individual genotype, j ($j = 1, 2, \cdots, N_{env}$) represents the index number of a sub-population testing environment, B_0 is the baseline payoff, B_{coop}, C_{coop}, C_{sig} are constants for the benefit of cooperation, cost of cooperation and cost of signaling, respectively, p_i is the basal signal production rate of the genotype i. The function of cooperation cost of the individual i in the sub-population testing environment j is defined as:

$$H_{C_{ij}} = \begin{cases} 1 & \text{if } \frac{1}{G} \sum\limits_g^G \frac{p_g N_j}{u} > S_{Th_g} \\ 0 & \text{otherwise} \end{cases}, \tag{S4}$$

where G is the number of mixing genotypes in a sub-population, N_j is the local cellular density in the jth sub-population testing environment, and p_g and S_{Th_g} are the signal production rate and signal response threshold of the genotype g ($g = 1, 2, \cdots, G$) in the sub-population, respectively. Similarly, the function of cooperative benefit of the individual i in the sub-population testing environment j is defined as:

$$H_{B_{ij}} = \begin{cases} 1 & \text{if } \frac{1}{G} \sum\limits_g^G N_j H_{C_{gj}} > N_{Th} \\ 0 & \text{otherwise} \end{cases}, \tag{S5}$$

where N_{Th} is the cellular density threshold (defined as the median cellular density across all testing environments).

Computational model of quorum sensing with auto-regulation

The computational model of signal dynamics for quorum sensing with auto-regulation is given as below:

$$\frac{dS}{dt} = p \left(1 + r \frac{S}{K + S}\right) N - uS, \tag{S6}$$

where S is the local signal concentration, t is time, N is the local cell density, p is the basal signal production rate, r is the ratio of auto-regulation production to basal signal production, K is the half concentration value, and u is the signal decay rate. The equilibrium of Eq. (S6) is given by:

$$S^* = \sqrt{N^2 p^2 r^2 + 2N^2 p^2 r + N^2 p^2 - 2Nkpru + 2Nkpu + k^2 u^2}. \tag{S7}$$

Note that previous studies have indicated the choice of Hill function exponent to be 2 [1, 2]. However, for the purpose of computational convenience, we used 1 as the Hill function exponent, which can lead to a close form solution, Eq. (S7). When invoking the auto-regulation mechanism, the individual genotype's overall cooperation payoff across all testing environments is assessed as follows:

$$F_i = B_0 + B_{coop} \sum_j H_{B_{ij}} - C_{coop} \sum_j H_{C_{ij}} - C_{sig} \left(1 + r_i \frac{S_i^*}{K + S_i^*}\right) p_i, \tag{S8}$$

where i ($i = 1, 2, \cdots, N_{pop}$) represents an individual genotype, j ($j = 1, 2, \cdots, N_{env}$) represents the index number of a sub-population testing environment, B_0 is the baseline payoff, B_{coop}, C_{coop}, C_{sig} are constants for the benefit of cooperation, cost of cooperation and cost of signaling, respectively, p_i and r_i are the basal signal production rate and auto-regulation ratio of the genotype i, respectively, and $S_i^* = \sum_j \bar{S}_{ij}^* \Big/ N_{env}$ where $\bar{S}_{ij}^*$ is defined in Eq. (S9). The function of cooperation cost of the individual i in the sub-population testing environment j is defined as:

$$H_{C_{ij}} = \begin{cases} 1 & \text{if } \bar{S}_{ij}^* = \frac{1}{G} \sum_g^G S_{gj}^* > S_{Th_g} \\ 0 & \text{otherwise} \end{cases}, \tag{S9}$$

where G is the number of mixing genotypes in a sub-population, S_{gj}^* (calculated by Eq. (S7)) and S_{Th_g} are the equilibrium signal concentration and signal response threshold of the genotype g ($g = 1, 2, \cdots, G$) in the sub-population, respectively. The function of cooperative benefit of the individual i in the sub-population testing environment j, and $H_{B_{ij}}$ is defined as the same as in Eq. (S5).

Adding noise to signal

To investigate how clonal populations cope with signal noise to sustain cooperation, we added noise to the equilibrium signal. In the simulations, the noise signal is drawn from a normal distribution with mean S^* and standard deviation $\kappa \cdot S^*$, i.e., $N(S^*, \kappa \cdot S^*)$, where S^* is the equilibrium signal calculated from Eq. (S2) or Eq. (S7) and κ is a constant indicating the strength of noise. Note we set all negative values for the noise signal to be 0.

Generating genetic mixing

In the evolution simulations where we varied the genetic relatedness, different numbers of mixing genotypes were introduced to form sub-populations to evaluate the overall cooperation payoff for each genotype in every generation. Unless specified otherwise, the actual number of mixing genotypes in each sub-population in every

generation was drawn from a zero-truncated Poisson distribution with the average being $\bar{G} = \lambda_G/(1-e^{-\lambda_G})$, where ($\lambda_G \in [0, 10]$; step size: 0.1). We define the clonal case ($G = 1$) when $\lambda_G = 0$ where exact one genotype will be selected to form the sub-population, i.e., no genetic mixing. Note that the number of sub-populations may be different in every generation due to the variation of mixing genotypes in each subpopulation.

Constructing constitutive cooperators

To investigate how decision making interact with social behaviors of cooperation, we compared the overall payoff of cooperation of individuals mediated by QS with those in the absence of collective control. Specifically, we constructed constitutive cooperators which do not have the ability to make social informed choices. In the clonal case, wild-type individuals will always cooperate. This will incur a penalty to each of such individuals for cooperating in wrong environments[1]. In the genetic mixing scenarios, the cooperative benefits of wild-type individuals will be shared evenly with all group members. In the simulations, all individuals were subject to mutation, switching from a wild-type to mutant, or mutant to wild-type depending on their own initial type. The actual number of replacement individuals was drawn from a Poisson distribution with the mean being 0.01. Note that mutant individuals will always reap the benefits of cooperation without paying for any cost. Formally, the overall cooperation payoff in the constitutive cooperation scenarios can be defined as:

$$F_i = \begin{cases} B_0 + B_{coop}\frac{N_{nev}}{2}P_{WT} - C_{coop}N_{nev} & \text{if indivudal } i \text{ is a wild-type} \\ B_0 + B_{coop}\frac{N_{nev}}{2}P_{WT} & \text{otherwise (mutant)} \end{cases}, \tag{S10}$$

where P_{WT} is the proportion of wild-type individuals in the sub-population with a group size G.

Introducing constitutive cheats

To challenge the quorum sensing system, in the simulations of invasion by a cheat phenotype, we replaced a certain number of individuals[2] chosen at random in the population pool with constitutive cheats in every generation at a certain rate. The constitutive cheat is defined as a genotype with a zero basal production rate and a maximum possible signal threshold. In other words, a constitutive cheat does not produce or respond to signal. The actual number of cheats introduced into each generation is drawn from a Poisson distribution with the mean being λ_{Cheat}. Note that the constitutive cheats are both immutable, which means they cannot be eliminated through mutation, and inheritable, which means their offspring are still cheats.

[1] Note that half of total testing environments are regarded as 'wrong' environments since we set N_{Th} as the median cellular density across all testing environments.

[2] Here, we only consider non-cheats. In other words, the existing cheats in the population pool will not be chosen.

Measuring phenotypic assortment of cooperative investment

To test if the auto-regulation mechanism could be explained by the generalized reciprocity theory, we recoded the mean value of cooperative investment[3] within each sub-population in the genetic mixing scenario where individuals are grouped into small collectives. We then plotted the group mean cooperative investment against individual cooperative investment. Finally, the regression line was fitted using the generalized linear model with a normal distribution. The slope of the regression line indicates the phenotypic assortment of cooperative investment. When the slope is high, the behaviors among individuals shifts closer to each other, investing more in cooperation. Otherwise, the behaviors of investment for cooperation vary among individuals.

Partitioning selection on cooperative investment

To further uncover the influence of the auto-regulation mechanism on cooperative behaviors in our evolution simulations, we employed the powerful conceptual framework of the Price equation to partition the selection on cooperative investment into both individual (within sub-populations) and group (between sub-populations) level [3]. The Price equation describes the change in the average amount of a trait (z) from one generation to the next (Δz) as a function of the covariance of between the fitness and the trait value among individuals ($\mathrm{Cov}(w_i, z_i)$), and the expected change in the amount of the trait value ($\mathrm{E}(w_i \Delta z_i)$) due to transmission error such as genetic drift, mutation bias, etc. The general form of the Price equation is given as below:

$$\bar{w}\Delta z = \mathrm{Cov}_i(w_i, z_i) + \mathrm{E}_i(w_i \Delta z_i), \tag{S11}$$

where z represents the trait cooperative investment, w_i is the number of offspring (fitness) produced by the individual i, $\bar{w}$ is the mean number of offspring produced, Δz_i represents the difference between the average z value among the individual i's offspring and i's own z value, $\mathrm{Cov}_i(\cdot)$ and $\mathrm{E}_i(\cdot)$ denote the expectation and covariance over all individuals i in the population respectively.

By introducing the genetic mixing in the simulations, individuals in the population have been assigned into small groups. We are able to further partition that selection based on cooperative investment to account for individuals that are nested within collectives. Specifically, we can expand Eq. (S11) by substituting it into the expectation term in its right hand side. Note that the groups that form each subpopulation g are the individuals ig. We can re-write the two-level Price equation as follows:

[3] The individual genotype's cooperative investment is simply defined as the number of sub-population testing environments where cooperation is turned on.

$$\bar{w}\Delta z = \mathrm{Cov}_g\left(\bar{w}_g, \bar{z}_g\right) + \mathrm{E}_g\left[\mathrm{Cov}_i(w_{ig}, z_{ig})\right] + \mathrm{E}_{ig}(w_{ig}\Delta z_{ig}), \tag{S12}$$

where $\bar{w}_g = \mathrm{E}(w_{ig})$ and $\bar{z}_g = \mathrm{E}(z_{ig})$. The first covariance term on the right hand side of the equation indicates the selection on cooperative investment at level of subpopulations (between-group selection), whereas the second expectation term captures the selection at individual level (within-group selection).

References

[1] Goryachev AB. Understanding Bacterial Cell-Cell Communication with Computational Modeling. Chemical Reviews. 2011;111(1):238–250.

[2] Pérez-Velázquez J, Quiñones B, Hense BA, Kuttler C. A mathematical model to investigate quorum sensing regulation and its heterogeneity in Pseudomonas syringae on leaves. Ecological Complexity. 2015;21:128–141.

[3] Price GR. Selection and Covariance. Nature. 1970;227:520–521.

[4] Brown SP, Johnstone RA. Cooperation in the dark: signalling and collective action in quorum-sensing bacteria. Proceedings of the Royal Society B: Biological Sciences. 2001;268(1470):961–965.

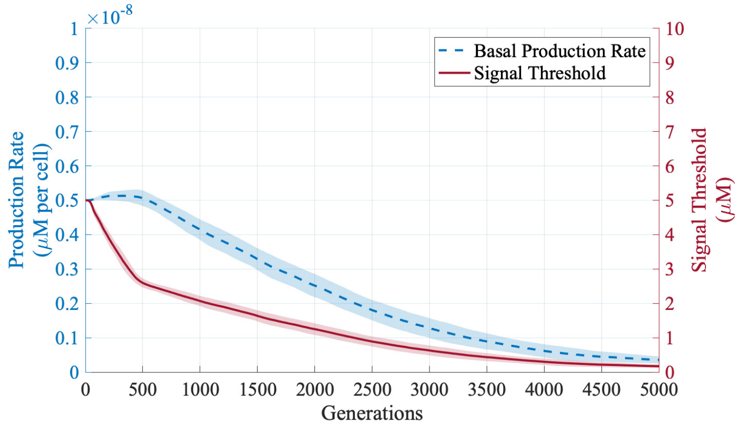

Figure S1. Evolution trajectories of basal production rate and signal threshold for high cost of signaling. We evolved $5,000$ initially identical genotypes for $5,000$ generations in a patchy, variable density environment (see *Main Text* Figure 6). In all simulations, the cost of cooperation was fixed and there was no auto-regulation. We used a fixed cost of signaling ($C_{sig} = 10^{10}$), and recorded the evolution trajectories of basal production rate and signal threshold. All reported results were averaged over 30 replications (shaded area indicates the standard deviation). The remaining parameters used in the simulations can be found in Table S1. Although the evolved traits were at equilibrium, the predicted transients are consistent with our previous mathematical model [4]. It should be noted that when $p = 0$, S_{Th} will stop evolving due to the cooperation collapse.

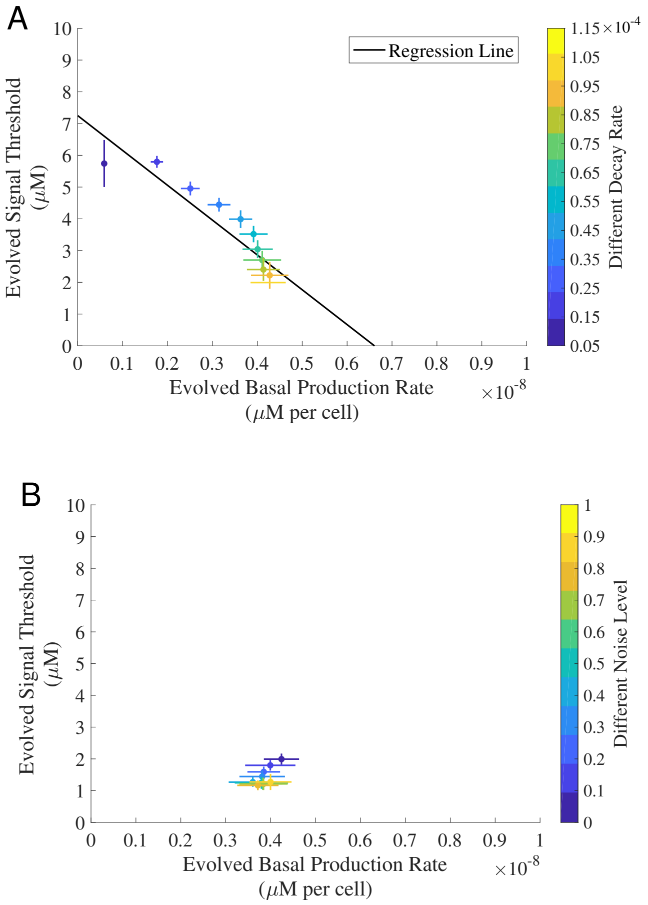

Figure S2. The evolved traits against signaling cost, decay rate and noise for QS-controlled cooperation. We evolved $5,000$ initially identical genotypes for $5,000$ generations. In all simulations, the cost of cooperation was fixed and there was no auto-regulation. Specifically, the population was evolved under three regimes: **(A)** Varying a range of decay rates ($u \in [5 \times 10^{-6}, 1.15 \times 10^{-4}]$; step size: 10^{-5}) with a fixed signaling cost and no noise ($\kappa = 0$), and **(B)** Varying levels of noise ($\kappa \in [0, 1]$; step size: 0.1) with a fixed signaling cost and a fixed decay rate ($u = 1.05 \times 10^{-4}$). Each dot represents the evolved mean results (averaged over the last 50 generations). The color-bars indicate different values of u and κ from low (dark blue) to high (dark red) in **(A)** and **(B)**, respectively. The solid black line in **(A)** is the regression line fitted using the generalized linear model with a normal distribution: $R^2 = 0.824$, F-test, $p = 4.443 \times 10^{-5}$. The horizontal and vertical error bars represent the standard deviation over 30 replications. The remaining parameters used in the simulations can be found in Table S1.

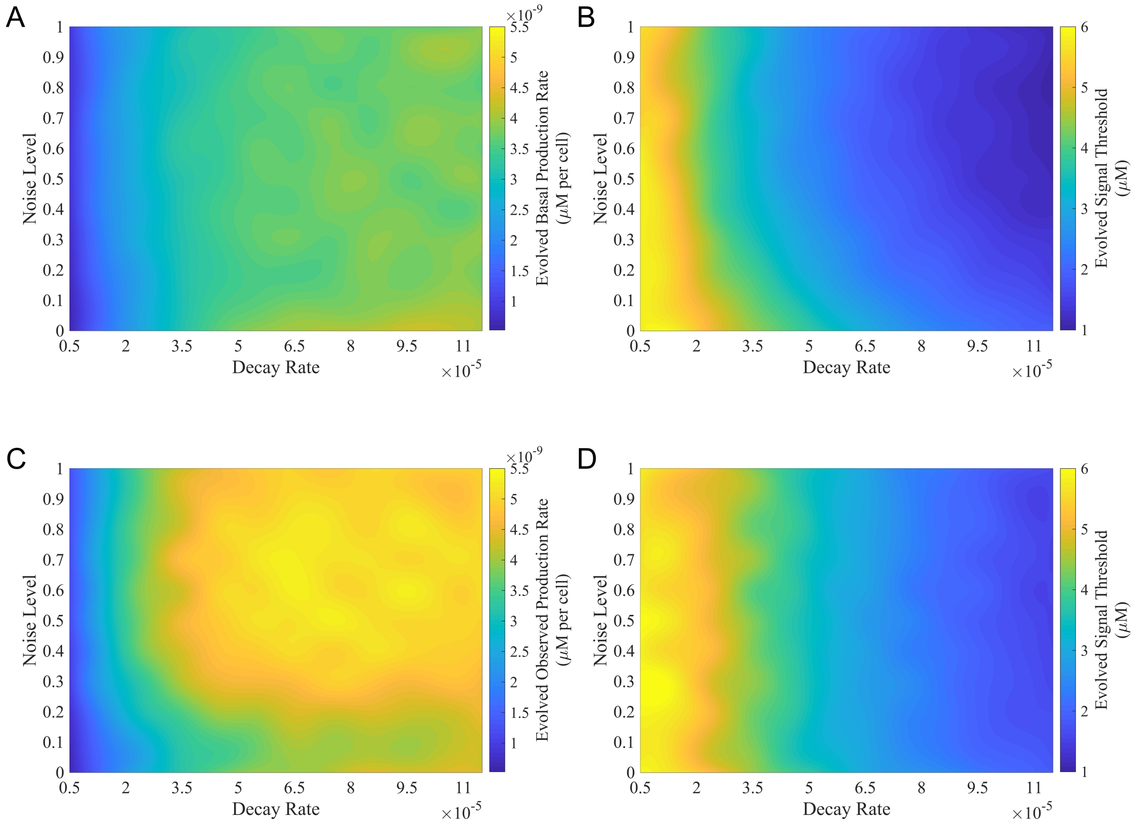

Figure S3. The evolved traits under different decay rates and levels of noise. We evolved 5,000 initially identical genotypes for 5,000 generations. In all simulations, the cost of cooperation and the cost of signaling were fixed. The decay rates, u, were varied in $[5 \times 10^{-6}, 1.15 \times 10^{-4}]$ (step size: 10^{-5}), and the levels of noise, κ, were varied in $[0, 1]$ (step size: 0.1). The results of basal and observed production rates were reported in **(A)** and **(C)** for QS with no auto-regulation and auto-regulation, respectively. The results of signal threshold were reported in **(B)** and **(D)** for QS with no auto-regulation and auto-regulation, respectively. All reported results were averaged over 30 replications. Note that surfaces were smoothed using the spline interpolation method. The remaining parameters used in the simulations can be found in Table S1.

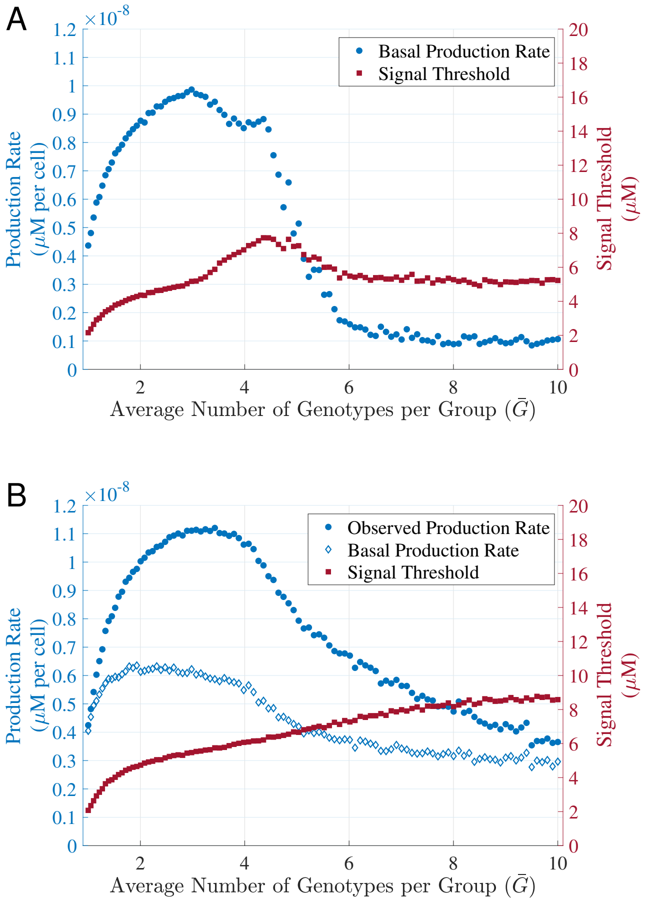

Figure S4. Evolved traits against genetic relatedness. We evolved $5,000$ initially identical genotypes for $5,000$ generations with no auto-regulation **(A)** and auto-regulation **(B)**, respectively. In all simulations, the cost of cooperation and the cost of signaling were fixed. Each dot represents the evolved mean results (averaged over the last 50 generations) for different average number of genotypes per group $\bar{G}$ ($\lambda_G \in [0, 10]$; step size: 0.1). The remaining parameters used in the simulations can be found in Table S1.

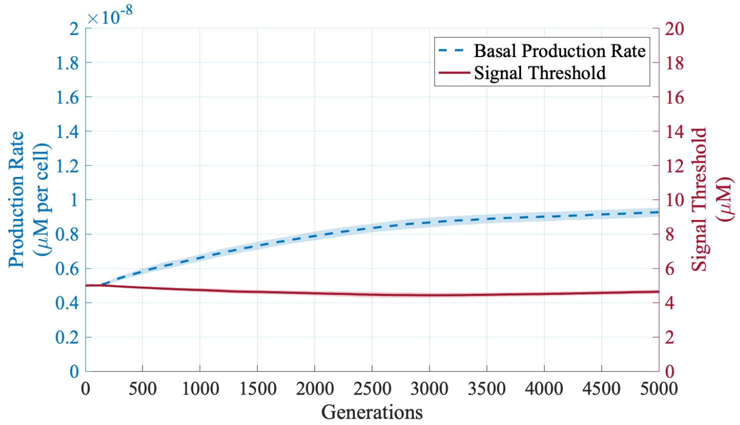

Figure S5. Evolution trajectories of traits for QS-controlled cooperation with no auto-regulation. For a population with an intermediate genetic mixing ($\lambda_G = 2$), we evolved $5,000$ initially identical genotypes for $5,000$ generations with no auto-regulation. We used a fixed cost of cooperation and a fixed cost of signaling, and recorded the evolution trajectories of basal production rate and signal threshold. All reported results were averaged over 30 replications (shaded area indicates the standard deviation). The remaining parameters used in the simulations can be found in Table S1.

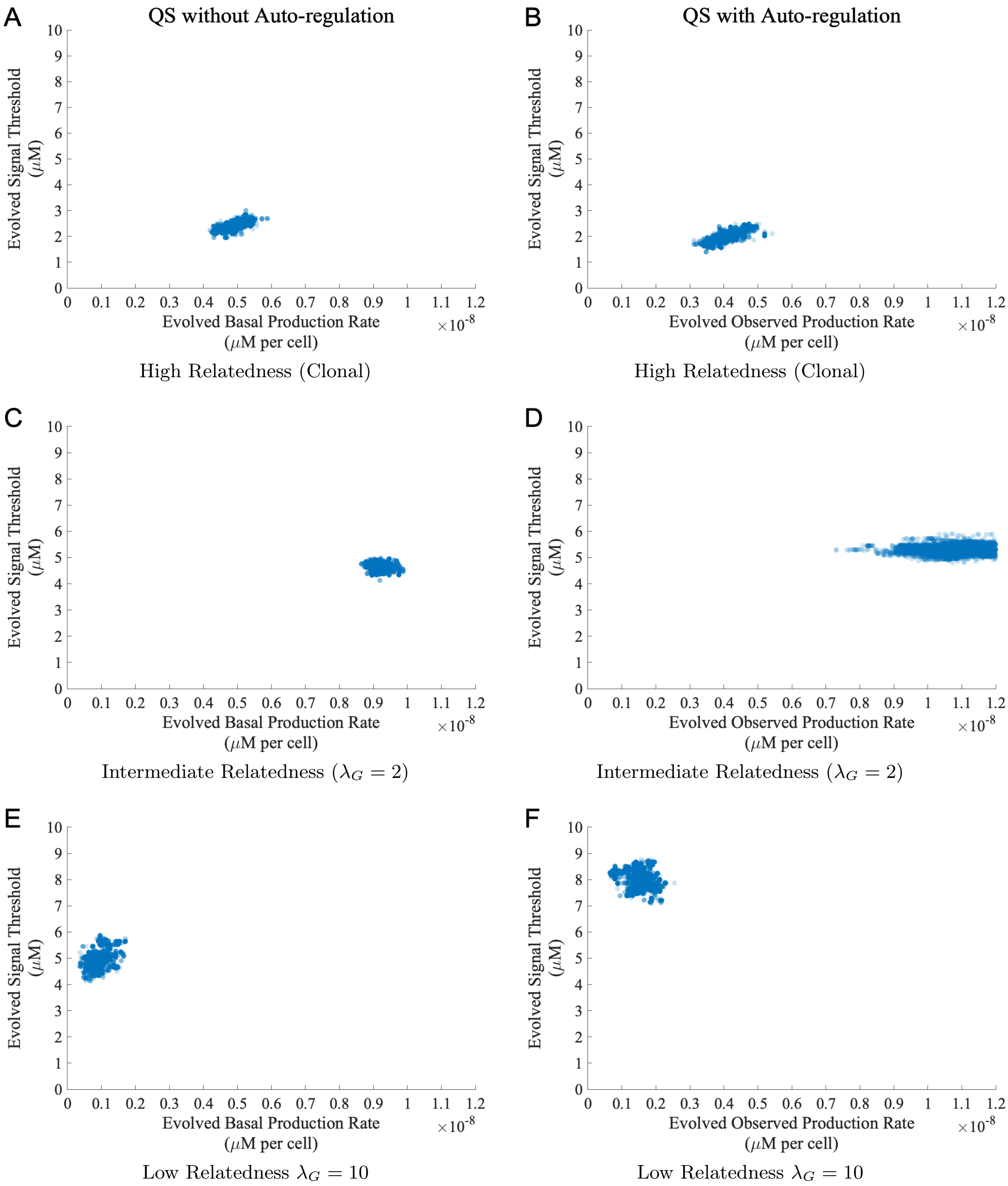

Figure S6. The diversity of traits in the evolved populations. We evolved $5,000$ initially identical genotypes for $5,000$ generations under high (clonal, $G = 1$), intermediate ($\lambda_G = 2$, $\bar{G} \approx 2.13$) and low ($\lambda_G = 10$, $\bar{G} \approx 10.00$) genetic mixing for both QS with no auto-regulation and auto-regulation, respectively. The trait values evolved after $5,000$ generations were reported in **(A)**, **(C)** and **(E)** for QS with no auto-regulation and **(B)**, **(D)** and **(F)** for QS with auto-regulation. All evolved trait values of $5,000$ individuals are plotted with blue dots. The color difference indicates the population density in the joint evolution of signal production (p) and signal threshold to response (S_{Th}). The remaining parameters used in the simulations can be found in Table S1. The full evolution timelapse can be found in *Supplemental Material*, Videos S1-S6.

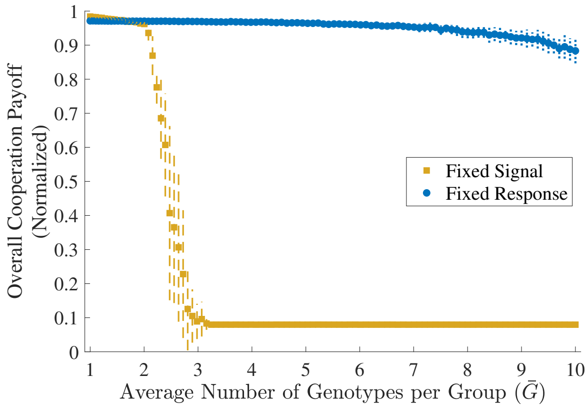

Figure S7. Overall cooperation payoff for fixed trait evolution. We used a fixed signal production rate of 0.5×10^{-8} and a fixed response threshold of 3 μM for QS-controlled cooperation without auto-regulation, respectively. In all cases, we evolved $5,000$ initially identical genotypes for $5,000$ generations. Each dot represents the evolved mean results (averaged over the last 50 generations) for different average number of genotypes per group $\bar{G}$ ($\lambda_G \in [0, 10]$; step size: 0.1). The vertical error bars represent the standard deviation of overall cooperation payoff over 30 replications. The remaining parameters used in the simulations can be found in Table S1.

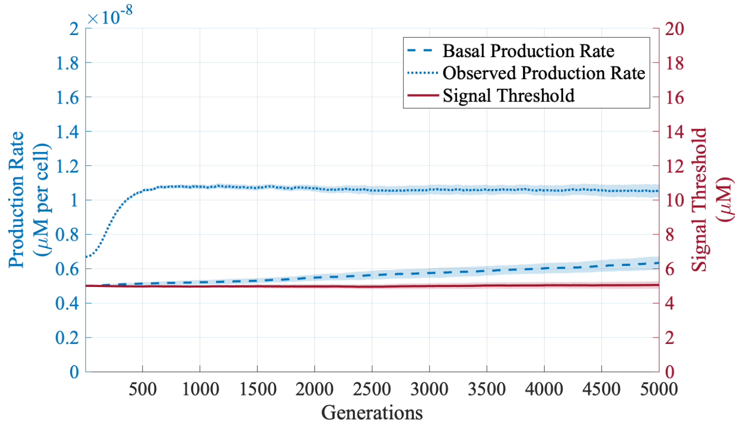

Figure S8. Evolution trajectories of traits for QS-controlled cooperation with auto-regulation. For a population with an intermediate genetic mixing ($\lambda_G = 2$), we evolved $5,000$ initially identical genotypes for $5,000$ generations with no auto-regulation. We used a fixed cost of cooperation and a fixed cost of signaling, and recorded the evolution trajectories of basal production rate, observed production rate and signal threshold. All reported results were averaged over 30 replications (shaded area indicates the standard deviation). Note that the basal production rate is slightly increasing which implies it is approaching the observed production rate over a longer evolution timescale. However, the observed production rate is largely converged. The remaining parameters used in the simulations can be found in Table S1.

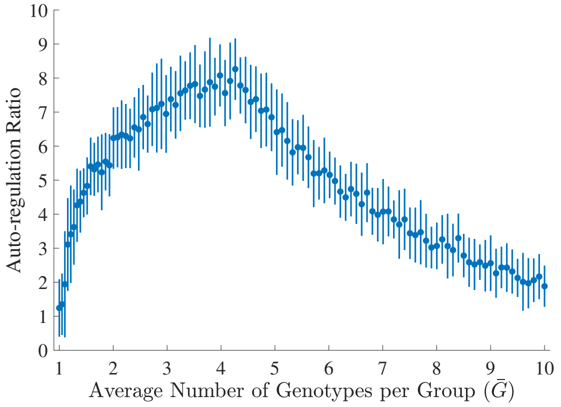

Figure S9. Evolved auto-regulation ratio against genetic relatedness. We evolved $5,000$ initially identical genotypes for $5,000$ generations with auto-regulation. In the simulations, the cost of cooperation and the cost of signaling were fixed. Each dot represents the evolved mean results (averaged over the last 50 generations) of auto-regulation ratio (r as in Eq. (S6)) for different average number of genotypes per group $\bar{G}$ ($\lambda_G \in [0, 10]$; step size: 0.1). The vertical error bars represent the standard deviation over 30 replications. The remaining parameters used in the simulations can be found in Table S1.

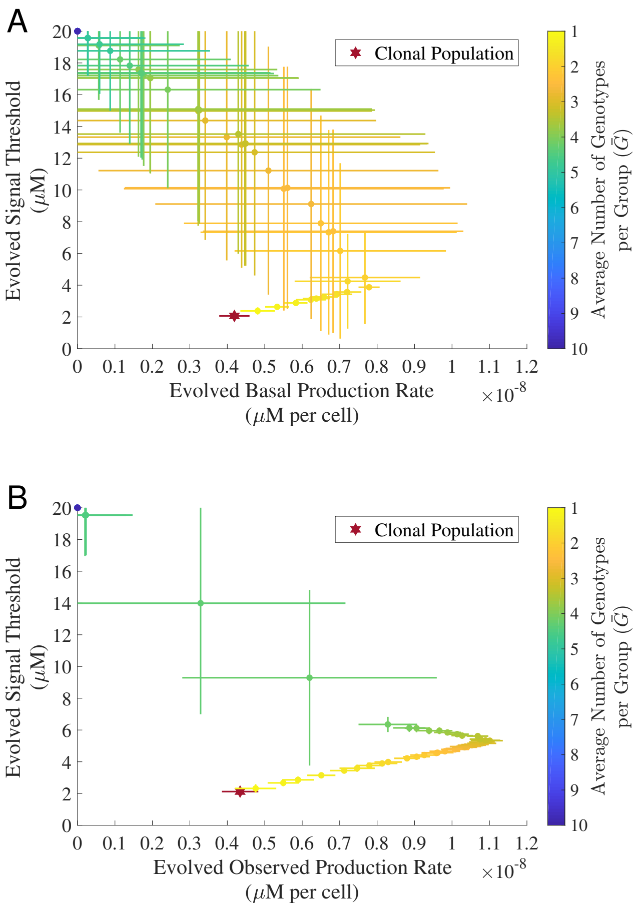

Figure S10. Invasion of constitutive cheats to the system with quorum sensing. We evolved $5,000$ initially identical genotypes for $5,000$ generations with no auto-regulation **(A)** and auto-regulation **(B)**, respectively. In all simulations, the cost of cooperation and the cost of signaling were fixed. A certain number of individuals (drawn from a Poisson distribution with $\lambda_{Cheat} = 0.1$) chosen at random were replaced with the constitutive cheats in every generation. Each dot represents the evolved mean results (averaged over the last 50 generations) for different average number of genotypes per group $\bar{G}$ ($\lambda_G \in [0, 10]$; step size: 0.1), indicated in the color-bar on the right. The star dot represents the clonal case when $G = 1$. The horizontal and vertical error bars on each dot represent the standard deviation of the results over 30 replications. The remaining parameters used in the simulations can be found in Table S1.

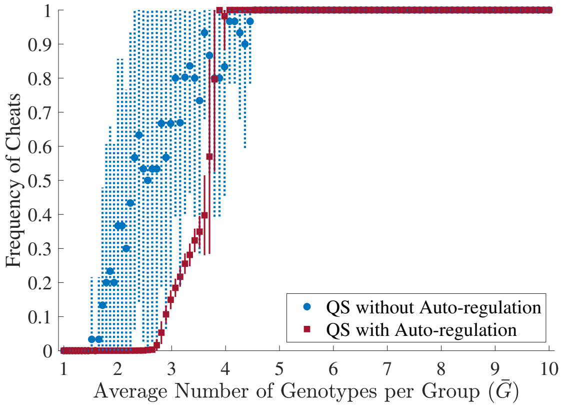

Figure S11. Comparison of frequency of cheats for the evolved system with or without auto-regulation. We evolved $5,000$ initially identical genotypes for $5,000$ generations with no auto-regulation **(A)** and auto-regulation **(B)**, respectively. In all simulations, the cost of cooperation and the cost of signaling were fixed. A certain number of individuals (drawn from a Poisson distribution with $\lambda_{Cheat} = 0.1$) chosen at random were replaced with the constitutive cheats in every generation. Each round dot (no auto-regulation) or square dot (auto-regulation) represents the evolved mean results (averaged over the last 50 generations) for different average number of genotypes per group $\bar{G}$ ($\lambda_G \in [0, 10]$; step size: 0.1). The vertical error bars represent the standard deviation over 30 replications. The remaining parameters used in the simulations can be found in Table S1.

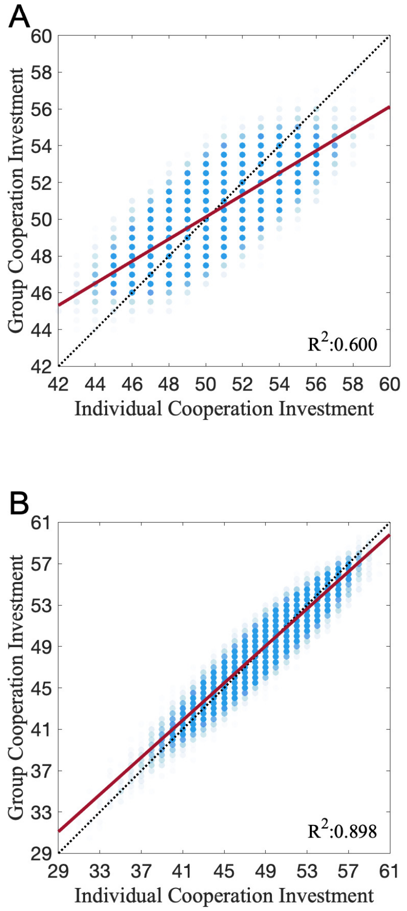

Figure S12. Regression analysis for individual and group mean investment for cooperation ($\boldsymbol{G = 2}$). For fixed costs of cooperation and signaling with the number of mixing genotypes $G = 2$, we collected $5,000$ same initial genotypes and evolved them for $5,000$ generations with no auto-regulation **(A)** and auto-regulation **(B)**. We recorded the individual and group mean investment for cooperation at the last generation over 100 replications. Each blue dot represents an individual's investment against its group mean investment. The red lines are the regression lines fitted using the generalized linear model with a normal distribution. The analysis of covariance shows there is a significant difference between the slope of no auto-regulation in **(A)** and the slope of auto-regulation in **(B)** (F-test, $p = 0.000$). The remaining parameters used in the simulations can be found in Table S1.

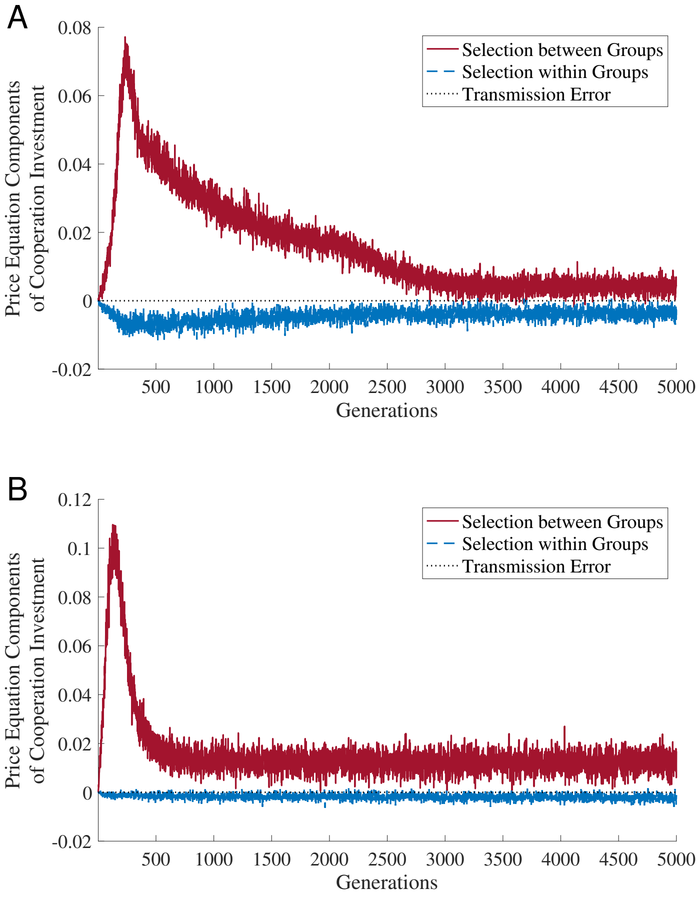

Figure S13. Selection on cooperative investment within and between groups ($G = 2$). We evolved $5,000$ initially identical genotypes for $5,000$ generations with no auto-regulation **(A)** and auto-regulation **(B)**, respectively. In all simulations, the cost of cooperation and the cost of signaling were fixed, and the number of mixing genotypes was fixed $G = 2$. We recorded the two-level Price equation components in every generation. The reported results were the average value over 100 replications. The remaining parameters used in the simulations can be found in Table S1.

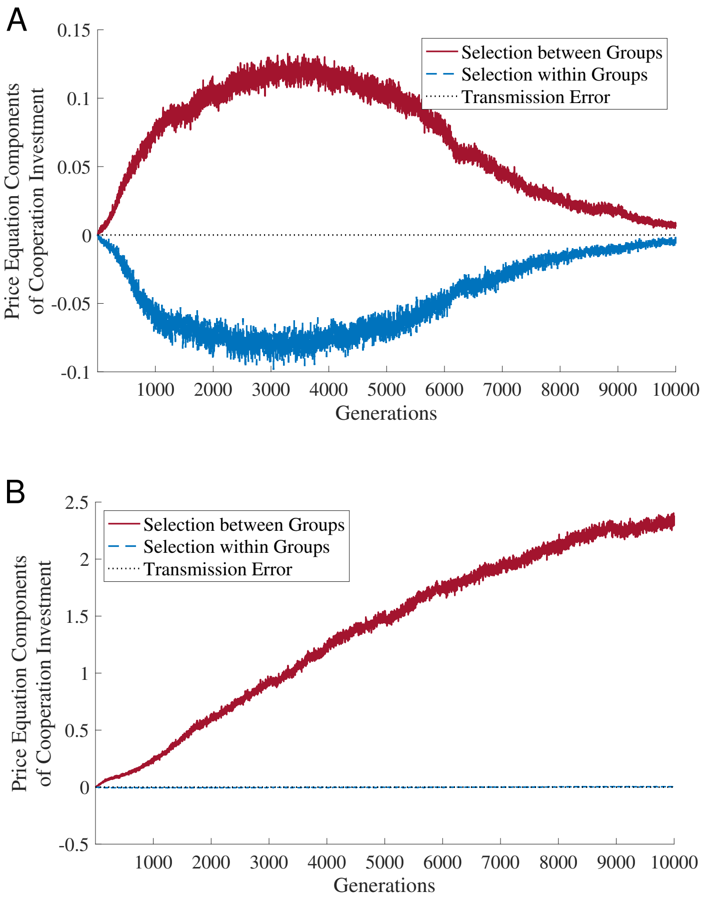

Figure S14. Selection on cooperative investment within and between groups ($G = 5$). We evolved $5,000$ initially identical genotypes for $5,000$ generations with no auto-regulation **(A)** and auto-regulation **(B)**, respectively. In all simulations, the cost of cooperation and the cost of signaling were fixed, and the number of mixing genotypes was fixed $G = 5$. We recorded the two-level Price equation components in every generation. The reported results were the average value over 100 replications. The remaining parameters used in the simulations can be found in Table S1.

Table S1. List of Model Parameters

Symbol	Description	Default Value
B_0	baseline payoff	100 FU[1]
B_{coop}	constant value for the benefit of cooperation	1.5 FU per CTE[2]
C_{coop}	constant value for the cost of cooperation	0.5 FU per CB[3]
C_{sig}	constant value for the cost of signalling	10^9 FU per μM
$Gen_{\max}$	the maximum of generations	5000
K	half concentration value	50 μM
G	number of mixing genotypes, unless otherwise specified, drawn from a zero-truncated Poisson distribution with the average being $\bar{G} = \lambda_G/(1-e^{-\lambda_G})$	1
N_{Th}	threshold of cellular density	50016 cells per μL
N_{env}	number of sub-population testing environments[4]	100
N_{pop}	population size	5000
SD_p	standard deviations for basal production rate	10^{-10}
SD_r	standard deviations for auto-regulation ratio	1
$SD_{S_{Th}}$	standard deviations for signal response threshold	0.1
$S_{Th_{\text{init}}}$	initial value for signal response threshold	5 μM
$S_{Th_{\min}}/S_{Th_{\max}}$	the minimum/maximum signal response threshold	0.001/20 μM
λ_p	mutate rate for basal production rate	0.01
λ_r	mutate rate for auto-regulation ratio	0.01
λ_G	parameter used in the zero-truncated Poisson distribution	0 to 10
$\lambda_{S_{Th}}$	mutate rate for signal response threshold	0.01
$p_{\min}/p_{\max}$	the minimum/maximum basal production rate	$0/2\times10^{-8}$ μMs^{-1} per cell
p_{init}	initial value for basal production rate	0.5×10^{-8} μMs^{-1} per cell
$r_{\min}/r_{\max}$	the minimum/maximum auto-regulation ratio	0.01/50
r_{init}	initial value for auto-regulation ratio	10
u	signal decay rate	10^{-4} μLs^{-1}
m	mass transfer rate	0 to 5×10^{-5} μLs^{-1}

[1] FU: fitness unit
[2] CTE: cooperative testing environment
[3] CB: cooperative behaviour
[4] The local cellular densities are evenly spaced within the range $10^{1.5}$ to 10^5 (cells per μL).
